# Supplementary material for: Identification of photoperiod-induced specific miRNAs in the adrenal glands of Sunite sheep (Ovis aries)
Source: Front Vet Sci. 2022 Jul 22;9:888207. doi: 10.3389/fvets.2022.888207 (PMC9354845; doi:10.3389/fvets.2022.888207)
Supplement: Supplementary file 1 [file Data_Sheet_1.ZIP › Supplementary file/miRDeep2 file.zip!/miRDeep2 file/Know_Structure/SP42A3/oar-mir-23a.pdf]

miRBase precursor	: oar-mir-23a
Total read count	: 61947
oar-miR-23a read count	: 31036
oar-miR-23b read count	: 30906
remaining reads	: 5

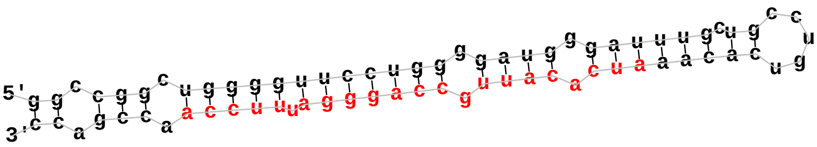

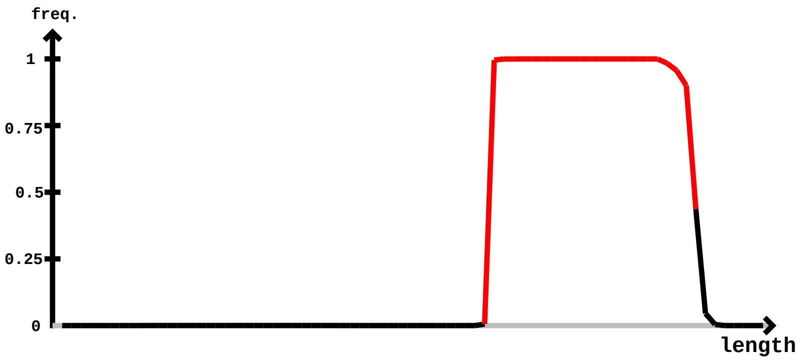


```
                                              oar-miR-23a
                                              oar-miR-23b
5'- ggccggcugggguuccuggggaugggauuugcugccugucacaaaucacauugccagggauuuccaaccgacc  -3'   exp
    ((.(((.((((((((((((.((((.((((((.((.....)))))))).)))).))))))).))))).))).))   reads   mm   sample
    .......ugggguuccuggggaugggauu............................................   1       0    seq
    ........gggguuccuggggaugggau.............................................   1       0    seq
    ........gggguuccuggggaugggauu............................................   1       0    seq
    ........gggguuccuggggaugggauuu...........................................   2       0    seq
    ...........................................aaucacauugccagggauu...........   4       0    seq
    ...........................................aaucacauugccagggauuu..........   10      0    seq
    ...........................................aaucacauugccagggauuuc.........   10      0    seq
    ...........................................aaucacauugccagggauuucc........   88      0    seq
    ...........................................aaucacauugccagggauuucca.......   55      0    seq
    ...........................................aaucacauugccagggauuuccaa......   4       0    seq
    ............................................aucacauugccagggau............   5       0    seq
    ............................................aucacauugccUgggauu...........   1       1    seq
    ............................................aucacauugccagggauu...........   489     0    seq
    ............................................aucacauugcUagggauu...........   2       1    seq
    ............................................aucacauugccagggauuu..........   844     0    seq
    ............................................aucacauugccagggauuuc.........   1730    0    seq
    ............................................aucacauugccagggauuucc........   14249   0    seq
    ............................................aucacauugccagggauuucca.......   12040   0    seq
    ............................................aucacauugccagggauuuccaa......   1263    0    seq
    ............................................aucacauugccagggauuuccaac.....   113     0    seq
    ..............................................ucacauugccagggauu..........   2       0    seq
    ..............................................ucacauugccagggauuuc........   8       0    seq
    ..............................................ucacauugccagggauuucc.......   41      0    seq
    ..............................................ucacauugccagggauuucca......   37      0    seq
    ..............................................ucacauugccagggauuuccaa.....   11      0    seq
    ..............................................ucacauugccagggauuuccaac....   17      0    seq
    ................................................cacauugccagggauuuc.......   1       0    seq
    ................................................cacauugccagggauuucc......   1       0    seq
    ..................................................acauugccagggauuucc.....   6       0    seq
    ..................................................acauugccagggauuucca....   5       0    seq
```
